# Fc-Mediated Functions of Porcine IgG Subclasses

**DOI:** 10.3389/fimmu.2022.903755

**Published:** 2022-06-09

**Authors:** Basudev Paudyal, William Mwangi, Pramila Rijal, John C. Schwartz, Alistair Noble, Andrew Shaw, Joshua E. Sealy, Marie Bonnet-Di Placido, Simon P. Graham, Alain Townsend, John A. Hammond, Elma Tchilian

**Affiliations:** ^1^Host Responses, The Pirbright Institute, Woking, United Kingdom; ^2^Medical Research Council (MRC) Human Immunology Unit, Weatherall Institute of Molecular Medicine, Radcliffe Department of Medicine, University of Oxford, Oxford, United Kingdom

**Keywords:** influenza monoclonal antibodies, porcine IgG subclasses, Fc functions, ADCC, CDCC, ADCP

## Abstract

The pig is an important agricultural species and powerful biomedical model. We have established the pig, a large natural host animal for influenza with many physiological similarities to humans, as a robust model for testing the therapeutic potential of monoclonal antibodies. Antibodies provide protection through neutralization and recruitment of innate effector functions through the Fc domain. However very little is known about the Fc-mediated functions of porcine IgG subclasses. We have generated 8 subclasses of two porcine monoclonal anti influenza hemagglutinin antibodies. We characterized their ability to activate complement, trigger cytotoxicity and phagocytosis by immune cells and assayed their binding to monocytes, macrophages, and natural killer cells. We show that IgG1, IgG2a, IgG2b, IgG2c and IgG4 bind well to targeted cell types and mediate complement mediated cellular cytotoxicity (CDCC), antibody dependent cellular cytotoxicity (ADCC) and antibody mediated cell phagocytosis (ADCP). IgG5b and IgG5c exhibited weak binding and variable and poor functional activity. Immune complexes of porcine IgG3 did not show any Fc-mediated functions except for binding to monocytes and macrophages and weak binding to NK cells. Interestingly, functionally similar porcine IgG subclasses clustered together in the genome. These novel findings will enhance the utility of the pig model for investigation of therapeutic antibodies.

## 1 Introduction

Amongst the five isotypes found in mammals, IgD, IgM, IgG, IgA and IgE, IgG is the most abundant antibody in serum, and is critical for humoral immunity. IgG has diverse subclasses, with different sizes, relative abundance, serum half-life, and ability for immune complex formation, complement activation, interaction with Fc receptor, effector functions, and placental transfer (1). IgG in humans, rats and mice has four subclasses. Human IgG1 and IgG3 are strong complement activators as are IgG2a and IgG2b in mice, while human IgG4 and mouse IgG1 are poor complement activators (2, 3). There are three orthologous IgG subclasses in cattle, goats, and sheep, and seven IgG subclasses in horses, although there is very little information about their effector functions (4–7). Chickens have only one antibody homologous to IgG referred to as IgY (8).

In pigs, six putative IgG subclasses were reported based on *IGHG* gene and cDNA sequencing, although recent genomic analyses indicate that all reported porcine *IGHG* genes can be classified into nine subclasses, several of which have been recently re-named *IGHG1*, *IGHG2a*, *IGHG2b*, *IGHG2c*, *IGHG3*, *IGHG4*, *IGHG5a*, *IGHG5b*, and *IGHG5c* (9–11). Of these, the *IGHG2* subclass genes are close paralogs of *IGHG1*, and the *IGHG5* genes are close paralogs of *IGHG4*. Importantly, the *IGHG2* and *IGHG5* genes are variably present and may not be present in any given porcine haplotype (10). We use this updated nomenclature throughout the remainder of this study.

Despite this recent development and some studies on porcine Fc receptors, knowledge of the function of IgG subclasses in the pig, a mammalian species with high agricultural and biomedical importance, is incomplete (12). Globally, 1.5 billion pigs are produced annually for pork production, which accounts for more than one quarter of total protein consumed worldwide. The demand for pork has led to an intensification of production, with farms often housing thousands of animals which can facilitate rapid pathogen transmission (13). African swine fever, salmonella, porcine reproductive and respiratory syndrome, foot and mouth disease and many other pathogens are major threats to pig production leading to substantial morbidity, mortality, loss of productivity and huge economic losses. Pigs are also natural hosts for the same subtypes of influenza A viruses as humans and are integrally involved in virus evolution with frequent interspecies transmissions in both directions. The emergence of the 2009 pandemic H1N1 virus (H1N1pdm09) illustrated the importance of pigs in the evolution of zoonotic strains. Pigs are anatomically, physiologically, and immunologically more similar to humans than small laboratory animals, such as mice and rats, and are commonly used to test vaccines and therapeutics in addition to being an excellent biomedical model and source of organ transplantation in humans (14–18). We have developed a swine influenza model to test vaccines, therapeutic monoclonal antibodies (mAbs) and mAb delivery platforms in a large natural host animal (19–23). We have shown that a strongly neutralizing human IgG1 mAb, 2-12C, against the hemagglutinin (HA) head, administered prophylactically to pigs reduced virus shedding and lung pathology after influenza challenge (20). To circumvent the anti-human Ig response, we developed porcine mAbs to pandemic H1N1pdm09, which recognized the same HA epitopes as human antibodies, and which increase the utility of the pig model in influenza virus research for evaluation of therapeutic mAbs and delivery platforms (22).

Although antigen binding by the Fab region of an antibody is crucial during antibody-mediated neutralization of a microbial pathogen, coupling Fab recognition to Fc-mediated effector function is essential in providing optimal protection *in vivo*. For example, Fc- FcγR interactions are required for protection *in vivo* by broadly neutralizing anti-HA and anti-neuraminidase influenza mAbs (24, 25). The FcγRs include both activating and inhibitory receptors, whose signals are balanced to regulate the outcome of an immune response. Mice express two low-affinity activating FcγRs on myeloid cells, FcγRIII and FcγRIV, as well as the low-affinity inhibitory FcγRIIb, which is widely expressed on hematopoietic cells. The biological activities of the mouse IgG subclasses are dependent on their relative affinities for the activating and inhibitory FcγRs. Mouse IgG2a is the most potent and interacts with the activating FcγRs, whereas IgG1 is the least activating. Similar activating FcγRIIa and FcγRIIIa and inhibitory FcγRIIb receptors exist in humans. Human IgG1 and IgG3 are strong complement activators and bind strongly to FcγRI, FcγRII and FcγRIII receptors, unlike IgG4 which does not activate complement and binds to FcγR with low affinity (1, 2). Recently the Göttingen minipig low-affinity *FCGR* genes were annotated and the distribution of porcine FcγRs on leukocytes was analysed (12). The binding properties of the porcine FcγRIa, FcγRIIa and FcγRIIb resembled those of the human orthologs, although porcine FcγRIIIa binds porcine IgG1a, but not human IgG1, while porcine FcγRIa, FcγRIIa, and FcγRIIb all bind human IgG1.

However, in the pig very little is known about the function of the Fc fragment of the different IgG subclasses. Based on sequence analysis, Butler et al, predicted that IgG3 is most likely to activate complement and bind to FcγRs with high affinity, in contrast to previous studies that showed both IgG1 and IgG2 can activate complement, IgG2 being more efficient than IgG1 (9, 26). This highlights a lack of consensus on key functions of porcine antibodies. Moreover, Fc functions such as complement-dependent cellular cytotoxicity (CDCC), antibody-dependent cellular cytotoxicity (ADCC), antibody-dependent cellular phagocytosis (ADCP), and binding of free and immune-complexed antibodies to leukocyte subsets are poorly characterized. Here, we have engineered 8 subclasses derived from two IgG1 porcine monoclonal antibodies recognizing different epitopes of the HA of H1N1pdm09 virus. We characterized their ability to activate complement, trigger cytotoxicity and phagocytosis of immune cells and assessed their binding to monocytes, macrophages and natural killer cells.

## 2 Materials and Methods

### 2.1 Production of Recombinant Pig Monoclonal Antibodies of Different IgG Isotypes

Generation of isotype switched versions of two porcine H1N1pdm09 HA specific mAbs, pb27 and pb39, followed a modification of the protocol described in our previous work (22). Briefly, the nucleotide sequences coding for pig *IGHG1* (AB699686), *IGHG2a* (AEMK02000452), *IGHG2b* (AK405792), *IGHG2c* (AB699687)), *IGHG3* (AB699686), *IGHG4* (AB699687), *IGHG5b* (AB699686), and *IGHG5c* (AB699686) were obtained from publicly available data and publications (11, 27). Synthetic genes (gblocks) for these sequences were purchased (Integrated DNA Technologies) and cloned into a vector derived from pNeoSec (28) kindly provided by Prof Raymond Owens, Protein Production UK (PPUK). The vectors encode the μ-phosphatase secretion leader and were modified to include the antibody V-region coding sequence cloning site in-frame to a downstream constant region coding sequence as previously described for expression of recombinant mouse antibodies (29). The V-region sequence replaces a region containing the lacZ promoter and lacZ alpha peptide flanked by KpnI (5’) and PstI (3’) allowing seamless in-frame joining to the pig constant region of the different IgG isotypes, and also blue and white selection of transformants.

The sequences of the variable parts of heavy and light chains of pb27 and pb39 were also purchased as synthetic genes (Integrated DNA Technologies) and cloned in-frame by In-Fusion cloning reaction using ClonExpress II One Step Cloning Kit (Vazyme) after linearizing the vectors with KpnI (5’) and PstI (3’). The 5’ end of the gblock had a 5’-15bp overlap specific for the leader sequence encoded in the expression vector, whereas the 3’ end contained a 15bp sequence identical to the 5’ end of the constant region of the respective construct thereby generating an overlap with the expression vectors on both ends suitable for In-Fusion cloning.

The heavy and light chain infusion reactions were transformed into high-efficiency Stellar Competent Cells (E. coli HST08 strain) (Takara). Three colonies from each transformation were picked and inoculated in 3 ml LB + Kanamycin overnight aerobically at 37˚C on an orbital shaker. Plasmid DNA was isolated with the QIAprep Spin Miniprep Kit (Qiagen, UK) and submitted to Sanger sequencing to ensure the sequences were correct.

The plasmid constructs of the heavy chain and their corresponding light chains were transiently co-transfected into Expi293F™ cells (Gibco™ A14527) using Polyethylenimine (PEI) MAX^®^ (Polysciences). Following 3 days of incubation, the cell culture supernatant was harvested and purified by Protein G chromatography (Cytiva 17040501). The pH neutralised eluate was dialysed in PBS and the concentration of the purified mAb was determined by A280/1.4 to be 1.8 mg/ml.

### 2.2 Selection of Virus Escape Variant With pb39

Antibody (30 µg) was incubated together with 0.5 mL X-179A virus (~5 x 10^6^ TCID_50_), for 1-2 h in room temperature. Virus-antibody mix was then transferred to a MDCK-SIAT1 cell monolayer in a 6-well plate. After 40 min, 1 µg/mL TPCK-trypsin was added to make final volume to 3 mL. ﻿Cells were incubated in humidified incubator at 37°C and 5% CO_2_. Virus in the culture supernatant was harvested after 48 hours. Second selection was performed using 0.5 mL of passage 1 virus in presence of antibody. To confirm the selection of escape variant, hemagglutinin inhibition assays (HAI) were performed with the higher concentration of antibody. Wildtype virus was passaged without antibody alongside the experiment. The HA and neuraminidase (NA) genes of the escape variant virus population were sequenced using RT-PCR and Sanger sequencing. The sequences were compared with the wild-type sequence. K209T was identified as a tissue culture adaptive substitution that was identified in both wild-type and pb39-escape viruses. Pb39-escape viruses showed an additional G53D mutation in HA1. No mutations were identified in NA genes.

### 2.3 Binding to MDCK-HA

Madin–Darby canine kidney–2,6-sialyltransferase cells stably expressing HA MDCK-SIAT1-HA from Influenza A H1N1 A/Eng/195/2009 (MDCK-HA) were used to determine the binding activity of the recombinant mAbs of different isotypes. Monolayers of overnight cultured MDCK-HA cells were washed and incubated with 50 µL of serially diluted mAbs for 30 min at room temperature (RT). Cells were washed and incubated with rabbit anti-pig IgG(H+L)-HRP antibody (Invitrogen) for 1h at RT. After a further wash, 50 µL 3,3’,5,5’-tetramethylbenzidine (TMB) substrate was added, and the reaction was stopped with 50 µL of 1M sulphuric acid. The absorbance was measured at 450 and 630nm on an ELx808 BioTek plate reader (BioTek).

### 2.4 Microneutralization

A microneutralization (MN) assay was used to determine the neutralising concentrations of the mAbs. Briefly, viruses were titrated to give a plateau expression of nucleoprotein (NP) in 96-well flat-bottomed plates. Viruses and mAbs were separately diluted in virus growth medium (VGM) containing DMEM, 100 IU/ml penicillin, 100 µg/mL streptomycin and 0.1% bovine serum albumin (BSA). mAbs were serially diluted 1:2 in 50µl. Fifty microliters of the diluted virus was added to 50 µL mAb and incubated for 1 h at 37°C. 100 µL of 3 × 10^4^ MDCK-SIAT1-HA cells were added to the wells containing the virus and mAb mixtures and incubated overnight at 37°C. The media was removed, and the monolayer was fixed with 4% paraformaldehyde, permeabilized with Triton-X100 and stained with mouse anti-NP IgG1 (clone AA5H, Bio-Rad Antibodies) followed by goat anti-mouse HRP (DAKO) antibody. TMB substrate was added and incubated for 2-5 min and the reaction stopped with 50 uL of 1M sulphuric acid followed by absorbance measurement as above. The neutralizing concentration of mAbs was defined as the concentration that caused a 50% reduction in NP expression.

### 2.5 Biolayer Interferometry

BLI was carried out on an Octet R8 instrument (Sartorius) to determine protein-protein interaction kinetics between mAbs and influenza virus. All steps were performed with the sample plate incubated at 30°C and agitated at 1,000 rpm. HBS-EP buffer (Teknova) was used to dilute reagents and hydrate biosensors during the baseline steps. The virus was diluted to a concentration of 0.8 nM for pb27 and 1.6nM for pb39 IgG in HBS-EP buffer with oseltamivir carboxylate (Roche), and zanamivir (Merck). Briefly, biotinylated anti-swine IgG (Merck) was immobilised onto streptavidin biosensors (Sartorius) at 50 nM for 300 s. The recombinant pb27 and pb39 isotypes were loaded onto the immobilised anti-swine IgG at 0.75 nM for 300 s. The binding between mAbs and influenza virus at 0.8, 0.4, 0.2, 0.1, 0.05, 0.025, 0.0125 and 0 nM for 3600 s was then performed followed by dissociation for 3600 s. Data were aligned to the average of the last 5 s of the baseline, interstep corrected to dissociation and Savitsky Golay filtering was applied. Data were fitted using a 1:1 model and an R^2^ of >0.99 was achieved. Data analysis was conducted using the Octet Analysis software version 12.2.1.23 (Sartorius).

### 2.6 Complement Dependent Cellular Cytotoxicity Assay

CDCC activity of mAbs and immune sera was measured using rabbit low-tox-H complement (Cedarlane Laboratories). An in-house pig complement was also made from coagulated serum from naive pigs, pre-adsorbed on MDCK cells expressing HA (MDCK-HA) for 1 hr at 4°C followed by preservation at -80°C. MDCK-HA cells were suspended in AIM-V medium (ThermoFisher) at a concentration of 3 × 10^5^ cells/mL. Hundred microliter of cells were incubated with 50 µL per well of serially diluted pig mAbs or anti-Nipah virus G protein IgG1 mAb as negative control mAb for 15 min at RT and mixed with the pre-adsorbed complement (rabbit or pig) at a final concentration of 5% for 2 h at 37°C. Cells were spun at 420 × *g* for 4 min and 50 µL of supernatant was transferred to a flat bottom plate to which 50 µL of LDH-substrate (Roche Diagnostics) was added to measure the released lactate dehydrogenase (LDH) from lysed cells due to complement activation. The plate was immediately read with the kinetic protocol (8 reads, every 1 min) at a wavelength of 490-630 nm in a ELx808 BioTek plate reader (BioTek). The level of complement activation was expressed as percentage lysis of target cells compared to maximum lysis induced by the addition of 2% Triton-X100. To test the CDCC titre of immune sera, samples were serially diluted and incubated with overnight cultured MDCK-HA cells for 30 min at 4°C. Cells were washed with PBS and 5% pre-adsorbed rabbit complement was added for 2 h at 37°C. All the other steps were performed as outlined for the mAbs above.

### 2.7 NK-Cell Degranulation and Antibody Dependent Cellular Cytotoxicity

Degranulation on NK cells was performed using a modified flow cytometry based assay using surface mobilisation of CD107a as a readout. Pig PBMC were stimulated overnight with recombinant pig IL-2 (20 ng/mL) (Kingfisher Biotech, Saint Paul, MN), IL-12 (25 ng/mL) and IL-18 (100 ng/Ml) (R&D Systems, Minneapolis, MN). ELISA plates (Nunc, Maxisorp) were coated overnight at 4°C with 5 µg per well recombinant pHA protein (HA from A/England/195/2009 (30) protein in a carbonate-bicarbonate buffer. Following three washes, plates were blocked with 1% BSA in PBS for 1 hr at room temperature. Pig pb27 and pb39 IgG mAbs and anti-Nipah G protein IgG1 as negative control at 10 µg/ml were added to HA coated or uncoated wells for 1 hr at RT. Overnight stimulated PBMC were washed and resuspended in AIM-V medium and 1 × 10^6^ cells were added to the plates together with anti-CD107a-FITC at 4 µg/mL (IgG1, clone 4E9/11, Bio-Rad), brefeldin A (GolgiPlug, BD Biosciences) and monensin (GolgiStop, BD Biosciences) and incubated for 5 h at 37°C. Cells were washed and stained with live/dead near infra-red stain (ThermoFisher), anti-CD3ϵ (clone: BB23-8E6-8C8, BD Biosciences) and CD8α (clone: 76-2-11, BD Biosciences) for 15 min at RT. The cells were washed, fixed, and data was acquired on a MACSQuant 10 flow cytometer (Miltenyi Biotec). Data was exported and further analysed using the FlowJo software (Tree Star Inc).

For ADCC, PBMC were stimulated overnight with rp IL-2, IL-12 and IL-18 as above. MDCK-HA cells were suspended in AIM-V medium (ThermoFisher) at a concentration of 3 × 10^5^ cells/mL. A 100 µL of stimulated PBMC were incubated with 50µL of serially diluted pb27 IgG subclasses for 15 min at RT. Stimulated PBMC were added at a ratio of 20:1 PBMC to MDCK-HA and incubated for 5h at 37°C. Cells were spun at 420 × *g* for 4 min and 50 µL of supernatant was transferred to a flat bottom plate to which 50 µL of LDH-substrate (Roche Diagnostics) was added to measure the released lactate dehydrogenase (LDH) from lysed cells. The plate was immediately read with the kinetic protocol (8 reads, every 1 min) at a wavelength of 490-630nm in a ELx808 BioTek plate reader (BioTek). The level of cell cytotoxicity was expressed as percentage lysis of target cells compared to maximum lysis induced by the addition of 2% Triton-X100.

### 2.8 Antibody Dependent Cellular Phagocytosis

Porcine monocytes were enriched from fresh PBMCs by removing CD3ϵ positive cells using a MACS cell separation LD column (Miltenyi Biotec). The monocyte containing fractions were incubated with 50 ng/mL of human M-CSF (Miltenyi Biotec) for 6 days to differentiate them into macrophages. MDCK-HA target cells were fluorescently labelled with CFSE (ThermoFisher) following the manufacturer’s instructions and incubated with serially diluted pb27/pb39 IgG subclasses or anti-Nipah virus G protein IgG1 mAb as negative control for 30 min at RT. Differentiated macrophages were fluorescently labelled with CellTrace Violet (ThermoFisher) and added at an effector to target ratio of 20:1, in AIM-V medium at 2 × 10^6^ cells/mL. ADCP was determined after 3 h of culture by flow cytometry as described above measuring the percentage of live macrophages that contained target cells indicated as double positive for CellTrace Violet and CFSE.

### 2.9 Fc Binding

To study the interactions of the different pig IgG subclasses with porcine blood cells that natively express FcγRs, immune complexes were generated by incubation of 10 µg/mL of pb27 IgG subclasses and control porcine anti-Nipah virus G protein IgG1 mAb with 0.05 nM of H1N1pdm09 for 1 h at 37°C. Monocyte derived macrophages were produced as described above. NK cells, monocytes or macrophages were added to either free pb27 IgG subclasses or immune complexes and incubated for 1 h at 4°C. The cells were washed, stained with Live/Dead near infra-red stain and immune complex binding detected by staining with rabbit-anti pig IgG (H+L)-FITC (Thermofisher). Binding to NK cells, monocytes, and monocyte-derived macrophages was determined by co-staining with CD3^-^CD8α^+^ for NK cell, CD172a^+^CD14^+^ for monocytes and CD203^+^ for monocyte derived macrophages (**Table 1**).

**Table 1:** Antibodies used to define porcine leucocytes.

Ab	Clone	Isotype	Supplier
Anti-porcine CD3	BB23-8E6-8C8	Mouse IgG2a	BD Biosciences
Anti-porcine CD8a	76-2-11	Mouse IgG2a	BD Biosciences
Anti-porcine CD172a	74-22-15A	Mouse IgG2b	BD Biosciences
Anti-human CD14	REA599	Mouse IgG2a	Miltenyi Biotec
Anti-porcine CD203	PM18-7	Mouse IgG1	Bio-Rad Laboratories

## 3 Results

### 3.1 Epitope Identification and Neutralising Function of Two Porcine Anti-Influenza HA-Specific Antibodies

Two porcine anti-influenza HA-specific mAbs, pb27 and pb39, were previously identified by isolating and sequencing single antigen-specific B cells from H1N1pmd09 infected pigs and recombinant expression as porcine IgG1 (22). Recombinant pb27 recognizes the K130 site of HA, exhibits high neutralizing activity and binds strongly to MDCK cells expressing HA from H1N1pdm09 (MDCK-HA). In contrast, pb39 is poorly neutralizing and shows weak binding to MDCK-HA.

**Figure 1:**
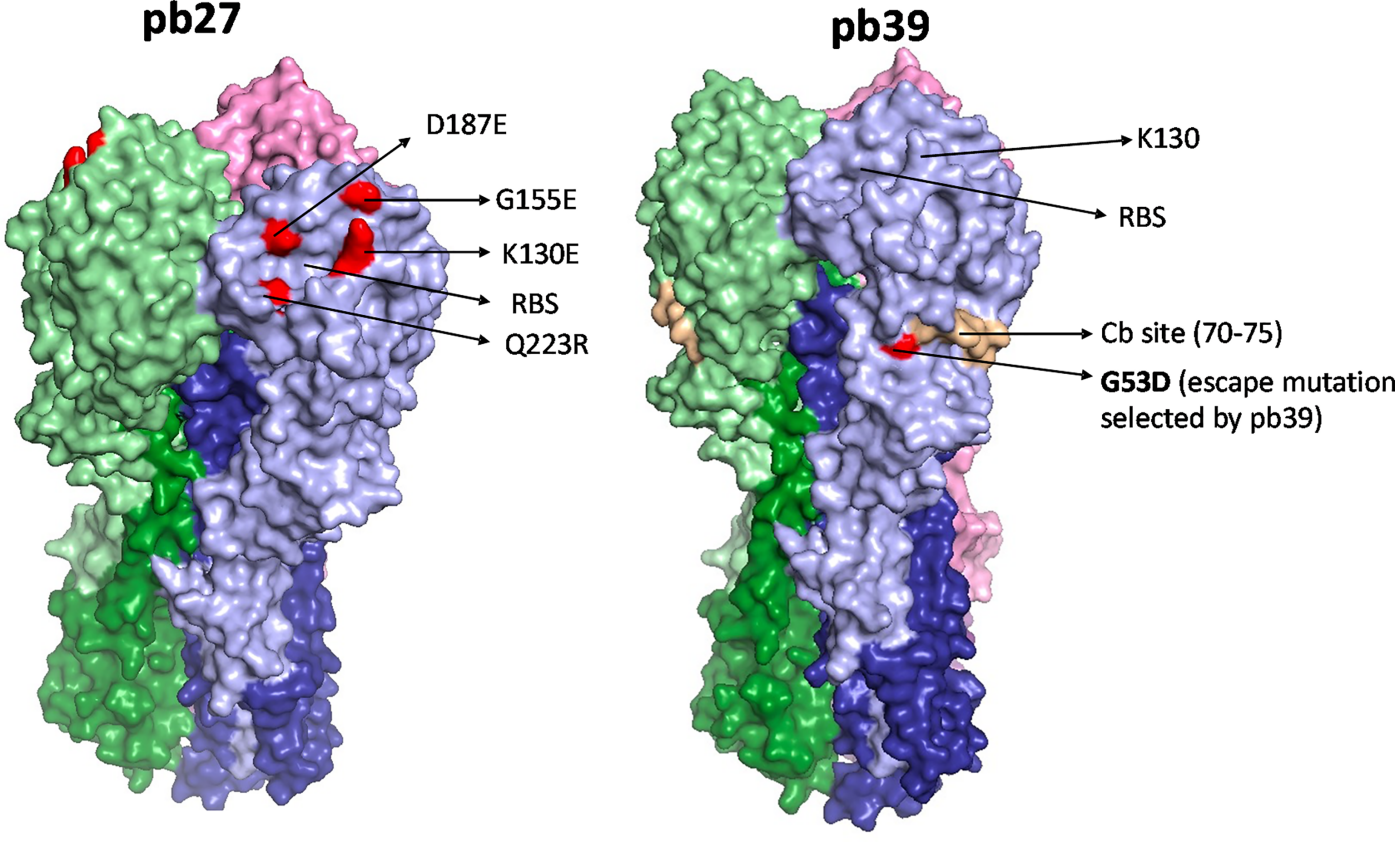
HA binding sites of pb27 and pb39. The receptor binding site is shown as a yellow circle and amino acid substitutions selected by antibodies in escape variants are shown as red. Mab pb27 recognized residues in antigenic Ca site surrounding the receptor binding site, namely K130E, G155E, D187E, and Q223R. Whereas, mAb pb39 selected substitution G53D in a Cb site. Substitutions were mapped with Pymol version 1.7 onto PDM:4M4Y.

To compare the binding activity and function of recombinant pb27 and pb39 it was first important to identify the location of pb39 epitope to determine any potential overlap. By selecting and sequencing an escape variant from the H1N1pdm09 vaccine virus X-179A (a PR8 reassortant virus with the HA and NA of A/California/07/2009) we identified a residue G53D substitution. G53 lies within the Cb site (one of the five antigenic sites described in the PR8 HA surface) which is further down the receptor binding site (RBS) (31) (**Figure 1**). Pb39 neutralised the X-179A viruses with substitutions in Sa site (K163E/Q) and in Ca site (K130E) suggesting it targets a non-overlapping epitope to other epitopes near the RBS (**Supplementary Figure 1**). Likely because of its distance from the RBS, pb39 is weaker in neutralization and HA inhibition, compared to dominant Sa or Ca2 site antibodies (22).

### 3.2 Porcine IgG Subclass Switching Does Not Significantly Alter the Binding or Neutralizing Activity of pb27 or pb39

**Figure 2:**
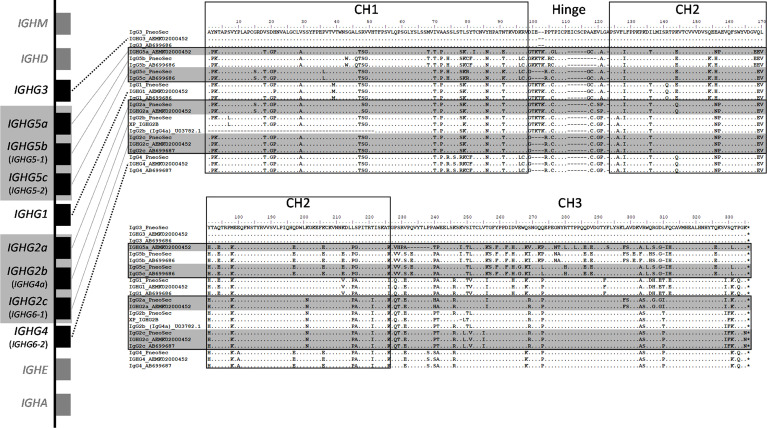
Genomic context and amino acid alignment of porcine IgG subclasses. The genomic context of the porcine IGH constant region is shown at left. The genes encoding the IgG2 and IgG5 subclasses regions are shaded gray, as these are variable in copy number and/or may not be present depending on an individual’s haplotype. Where applicable, old IgG subclass nomenclature is parenthesized and shown in smaller font below current nomenclature, which is based on Zhang et al., 2020 (10). The flanking genes that encode IgD, IgM, IgE, and IgA are in grey and shown for context. Presently used nomenclature is based on. Gene sizes and intergenic distances are not to scale. The amino acid alignments include the construct sequences used in the present study (“PneoSec”), and genomic sequences from the reference assembly, Sscrofa11 (AEMK02000452), and a previous Yorkshire bacterial artificial chromosome assembly (AB699686 and AB699687) (11). For IgG2b, supporting sequences are from a Xiang pig (XP_IGHG2B) as described by Zhang et al., 2020 and a partial Yorkshire cDNA sequence (U03782). Asterisks (*) at the end of the alignments indicate the termination codons.

**Figure 3:**
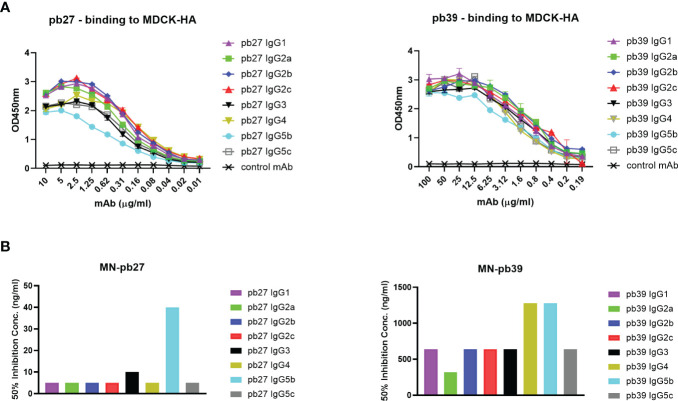
Binding and neutralizing activity of pb27 and pb39 IgG subclasses. **(A)** Binding activity of pb27 and pb39 IgG isotypes. **(B)** Neutralizing activity of pb27 and pb39 IgG subclasses. Anti-fluorescein human IgG1 (Absolute Antibody) was used as a control. The taller bars represent poorly neutralising antibodies. Representative data from 3 independent experiments shown.

Based on all the currently available high-quality genomic data, we selected eight IgG subclasses that represent all the known major pig genes or alleles (IgG1, IgG2a, IgG2b, IgG2c, IgG3, IgG4, IgG5b, and IgG5c (**Figure 2**). Recombinant chimeric pb27 and pb39 representing each subclass were then generated to characterize the leukocyte binding profile and Fc-mediated function of each subclass. To determine whether the IgG subclass influenced the binding or neutralizing activity of pb27 and pb39, we measured binding to MDCK-HA cells and their neutralization of H1N1pdm09. All pb27 IgG subclasses showed similar binding, except for pb27 IgG5b which showed a reduced level (**Figure 3A**). Correspondingly, pb27 IgG5b had reduced neutralizing activity (**Figure 3B**). All the pb39 subclasses required higher concentration for binding to MDCK-HA and microneutralization as previously shown, with pb39 IgG5b also showing slightly reduced binding and neutralization compared to the other pb39 subclasses (22).

To confirm if antibody binding affinity to HA is affected by subclass switching, we carried out biolayer interferometry (BLI) on an Octet R8 to provide better resolution. The KD for the pb27 IgG isotypes were similar, indicating that the affinity was not affected because of switching subclasses (range from 4.6 × 10^-12^ to 5.7 × 10^-12^M). The KD of different pb39 subclasses was greater (range 1.1. × 10^-9^ to 3.2 × 10^-11^M), indicating lower affinity (**Table 2**). Despite using substantially higher concentrations of HA, the response observed using BLI was lower for pb39 relative to pb27 (**Supplementary Figure 2A**) The lower levels of response were reflected in the association rates (K_a_), mean 1.4 × 10^5^ for pb39 and 3.3 × 10^6^ for pb27. Interestingly, the dissociation rates (K_dis_) were similar between pb27 and pb39 (mean 1.6 × 10^-5^ and 2.1 × 10^-5^) (**Supplementary Figure 2B**). These data suggest that the slower association rate for pb39, perhaps due to epitope inaccessibility has the greatest impact on affinity (32).

**Table 2:** Kinetic constants with standard error and fit parameters for pb27 and pb39 IgG subclasses.

mAb	Subclass (new nomenclat ure)	Subclass (old nomencla ture)	MN(ng/ml)	KD (M)	KD Error	K_a_(1/Ms)	K_a_Error	K_dis_(1/S)	K_dis_Error	X^2^	R^2^
**pb27**	IgG1	IgG1	5	5.6x10-12	1.5x10-14	2.9x106	1.7x103	1.6x10-5	4.4x10-8	29.969	0.99
	IgG2a	IgG2a	5	4.1x10-12	6.1x10-15	4.3x106	1.7x103	1.7x10-5	2.5x10-8	2.7416	0.99
	IgG2b	IgG4a	5	4.8x10-12	2.4x10-14	2.4x106	1.6x103	1.2x10-5	6.1x10-8	102.355	0.99
	IgG2c	IgG6.1	5	5.1x10-12	1.0x10-14	3.6x106	1.7x103	1.8x10-5	3.9x10-8	19.3179	0.99
	IgG3	IgG3	10	5.7x10-12	1.2x10-14	2.9x106	1.3x103	1.7x10-5	3.5x10-8	18.5426	0.99
	IgG4	IgG6.2	5	4.6x10-12	1.1x10-14	3.5x106	1.6x103	1.6x10-5	4.1x10-8	13.1264	0.99
	IgG5b	IgG5.1	40	5.7x10-12	3.5x10-14	2.8x106	3.3x103	1.6x10-5	9.8x10-8	52.5166	0.99
	IgG5c	IgG5.2	5	5.0x10-12	1.4x10-14	3.2x106	1.8x103	1.6x10-5	4.5x10-8	18.9703	0.99
**pb39**	IgG1	IgG1	640	1.2x10-10	7.1x10-13	1.1x105	2.6x102	1.3x10-5	7.1x10-8	27.0065	0.99
	IgG2a	IgG2a	320	1.4x10-10	1.8x10-12	1.2x105	1.1x103	1.8x10-5	1.6x10-7	17.4855	0.99
	IgG2b	IgG4a	640	1.1x10-9	4.9x10-11	2.5x104	1.1x103	2.7x10-5	1.5x10-7	15.1037	0.99
	IgG2c	IgG6.1	640	1.0x10-10	4.0x10-13	1.8x105	2.9x102	1.9x10-5	7.0x10-8	27.9447	0.99
	IgG3	IgG3	640	3.4x10-10	2.2x10-12	1.3x105	8.0x102	4.6x10-5	1.1x10-7	10.2786	0.99
	IgG4	IgG6.2	1280	2.0x10-10	8.3x10-13	9.0x104	2.2x102	1.8x10-5	5.9x10-8	37.2911	0.99
	IgG5b	IgG5.1	1280	1.2x10-10	1.6x10-12	8.8x104	5.1x102	1.0x10-5	1.3x10-7	64.9777	0.99
	IgG5c	IgG5.2	640	3.2x10-11	2.3x10-13	3.8x105	4.2x102	1.2x10-5	8.7x10-8	13.1969	0.99

MN, Microneutralization; KD, Dissociation equilibrium constant (affinity); K_a_, Association rate constant; K_dis_, Dissociation rate constant; X^2^,goodness of curve fitting; R^2^,Coefficient of determination.

Overall, these results confirmed that all subclasses of pb27 exhibited similar binding and neutralizing activity except IgG5b, which showed similar affinity as determined by BLI, but reduced binding and microneutralization. All pb39 subclasses showed similar activity and binding except for a marginally reduced binding and microneutralization observed for IgG5b.

### 3.3 Binding of pb27 IgG Subclasses to Monocytes, Macrophages, and Natural Killer Cells

**Figure 4:**
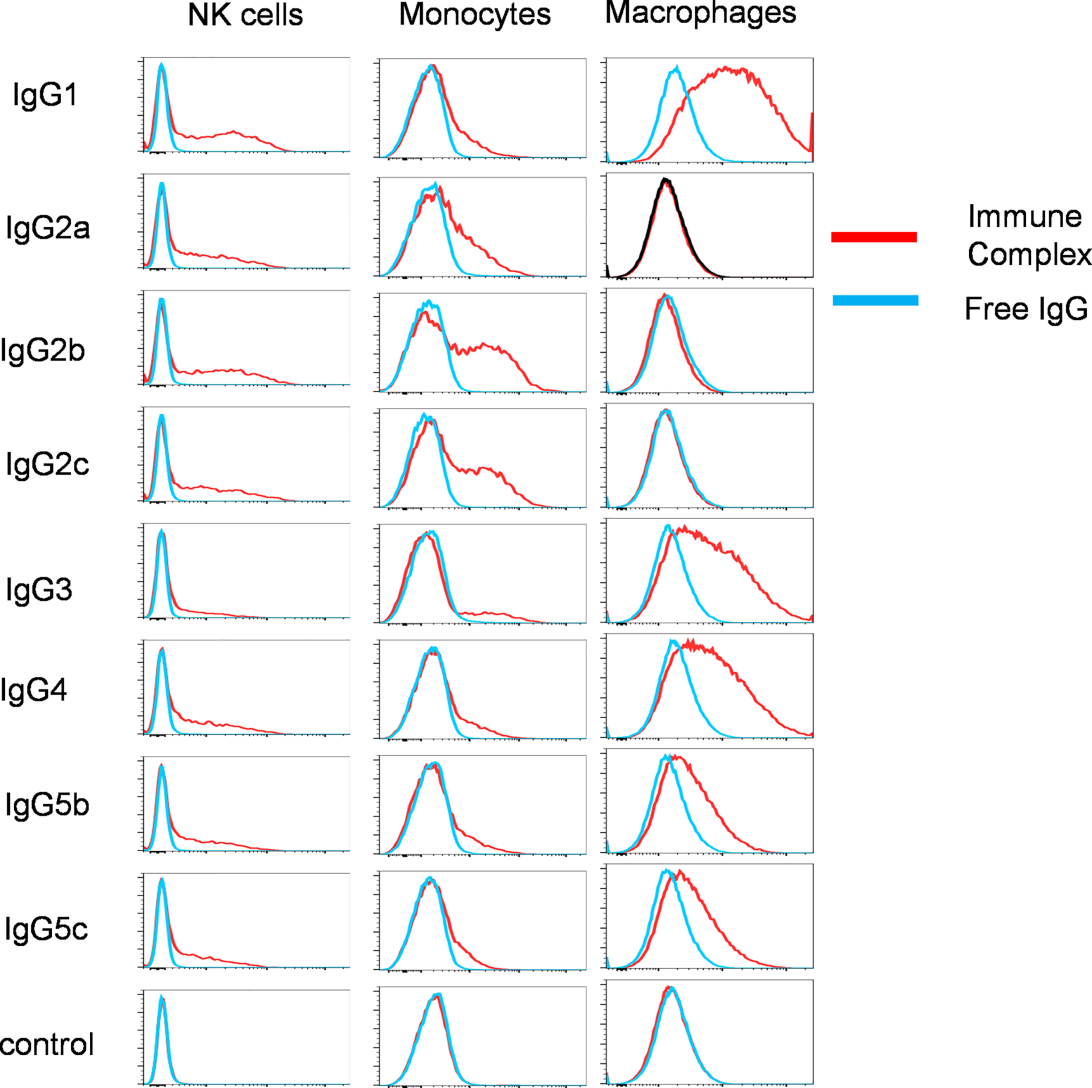
Binding of pb27 IgG subclasses to leucocytes. Binding of free (blue) and H1N1pdm09 virus immune complexed pb27 IgG subclasses (red) to NK cells, monocytes and macrophages. Anti-Nipah virus G protein IgG1 mAb was used as a control. Representative profiles from 3 experiments are shown.

To examine the IgG binding potential to immune cell populations, we determined the binding of free IgG or virus associated-IgG immune complexes with porcine leucocytes. We focussed solely on pb27 due to the stronger binding profile. All IgG subclasses were incubated with H1N1pdm09 virus to form immune complex and assayed for binding to NK cells, monocytes, and monocyte-derived macrophages (flow cytometry gating strategy shown in **Supplementary Figure 3**). Immune complexes with pb27 IgG1, IgG2a, IgG2b, IgG2c, IgG4 IgG5b, IgG5c bound to NK cells, but IgG3 showed minimal binding (**Figure 4**). All IgG subclasses bound to monocytes, which have been shown to express both FcγRIIa/b and FcγRIII (12), with IgG1, IgG2b, IgG2c, IgG3, and IgG4 showing the strongest binding. Monocyte-derived macrophages bound the fewest subclasses, IgG1, IgG3, IgG4, IgG5b and IgG5c with no measurable binding with IgG2a, IgG2b and IgG2c (**Figure 4**).

### 3.4 Functional Profiling of IgG Subtypes

#### 3.4.1 Complement Dependent Cellular Cytotoxicity

**Figure 5:**
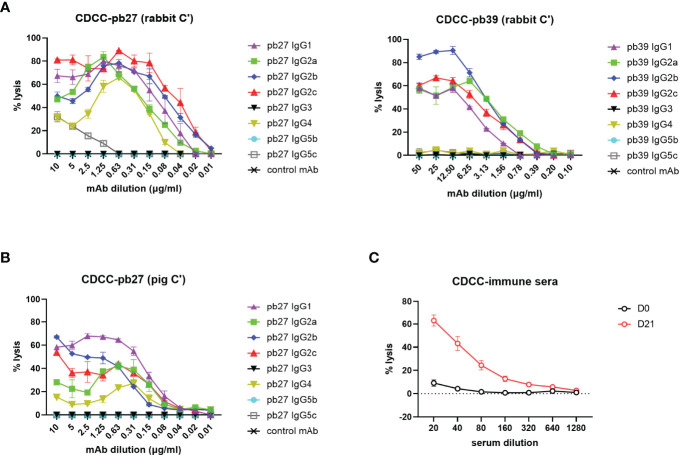
Complement dependent cytotoxicity mediated by pig IgG subclasses. **(A)** CDCC activity of IgG subclasses were measured on MDCK-HA incubated with a serial dilution of pb27 and pb39 IgG subclasses in the presence of rabbit complement and **(B)** pig complement. Error bar represents the SD of 3 independent experiments. **(C)** CDCC activity of immune sera was measured in the presence of rabbit complement, error bar represents SEM, n=6.

A complement-dependent cellular cytotoxicity (CDCC) assay was used to study the Fc-dependent ability of the pig IgG subclasses to trigger complement component C1q leading to target cell lysis. We used both autologous pig complement and the more potent rabbit complement to lyse MDCK-HA target cells. Pb27 subclasses, IgG1, IgG2a, IgG2b, IgG2c and IgG4 showed strong rabbit complement activation, while IgG5c induced a weaker response and IgG3 and IgG5b showed no complement activation (**Figure 5A**). Although the overall response was lower, an almost identical pattern of lysis was seen when using pig complement: IgG subclasses IgG1, IgG2a, IgG2b, IgG2c and IgG4 showed strong lytic activity (**Figure 5B**). For pb39, subclasses IgG3, IgG4, IgG5b and IgG5c did not activate complement (**Figure 5A**). We also demonstrate the suitability of this assay for assessing complement-dependent function of immune serum from H1N1pmd09 infected pigs at 21 days post infection, which induced 60% lysis at 1:20 dilution with rabbit complement (**Figure 5C**).

These data demonstrated that not all subclasses activated rabbit or pig complement and IgG3 and IgG5b were unable to mediate lysis with either complement. The extent of lysis and complement activation was also affected by the specificity and affinity of the mAb used, with pb39 subclasses showing weaker lysis relative to pb27 subclasses.

#### 3.4.2 Antibody-Dependent Cellular Cytotoxicity

**Figure 6:**
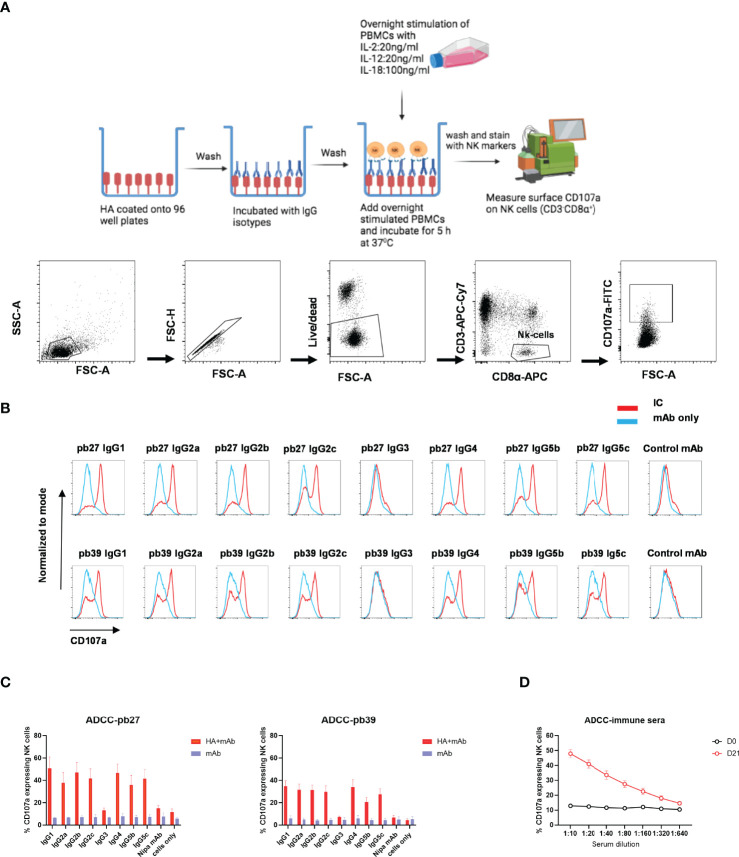
NK cell degranulation activity of pb27 and pb39 IgG subclasses. **(A)** Schematic of the assay setup (figure was created with Biorender.com) and flow cytometry gating strategy for NK cells defined as CD3^-^CD8α^+^. **(B)** Histogram normalized to mode showing the degranulation by pb27 and pb39 subclasses complexed with HA (red) and IgG only (blue). Anti-Nipah virus G protein IgG1 mAb was used as a control. **(C)** Bar chart showing degranulation by IgG complex with HA (red) or IgG only (blue). The error bars represent the SEM, n=4. **(D)** Assessment of degranulation titer of immune and naïve sera. Error bar represents the SEM, n=6.

NK cell degranulation based on CD107a mobilisation is an indicator of cytolytic activity (33). In this study, peripheral blood mononuclear cells (PBMC) were stimulated with recombinant porcine (rp)IL-2, rpIL-12 and rpIL-18 to differentiate NK cells into cytotoxic effectors (34). Porcine NK cells were defined as CD3^-^ CD8α^+^ (**Figure 6A**). Recombinant HA was immobilized on microtiter plate to enable FcR crosslinking by the pb27 or pb39 subclasses resulting in the mobilization of CD107a onto the cell surface of activated NK cells as detected by flow cytometry (**Figure 6A**). All pb27 and pb39 IgG subclasses except IgG3 induced CD107a onto the cell surface. The levels of CD107a were higher for pb27 than pb39 consistent with antibody affinity (**Figures 6B, C**). Porcine immune serum obtained 21-day post H1Npdm09 infection mediated a robust dose-dependent CD107a response compared to control serum (**Figure 6D**). Antibody dependent cellular cytotoxicity (ADCC) was also measured using MDCK-HA cells as targets and activated PBMC as effectors (**Supplementary Figure 4A**). Although there was more variability in this assay the results were consistent with IgG3 and IgG5b displaying the weakest, IgG2a, IgG2b and IgG5c moderate and IgG1, IgG2c and IgG4 the highest lytic activity. Day 21 post infection serum also mediated ADCC (**Supplementary Figure 4B**).

These data show that IgG3 was unable to mediate NK cell degranulation or lysis of target cells, although all other subclasses showed some activity.

#### 3.4.3 Antibody-Dependent Cellular Phagocytosis

**Figure 7:**
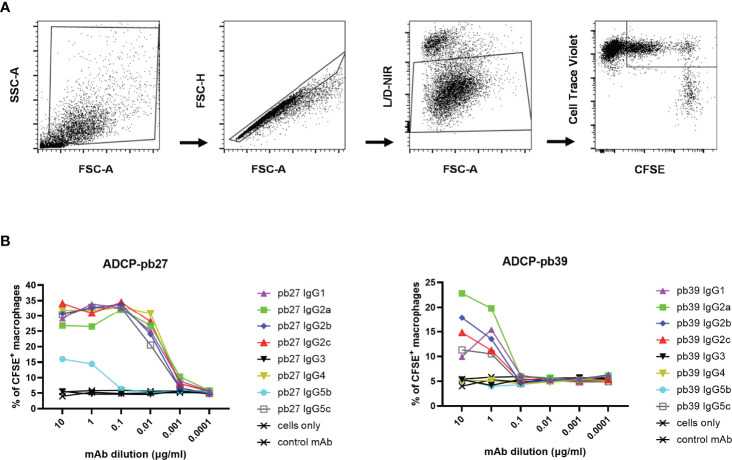
Phagocytic activity induced by pb27 and pb39 IgG subclasses. **(A)** Gating strategy for flow cytometry analysis. MDCK-HA cells and differentiated macrophages were stained with CFSE and CellTrace Violet respectively and near IR live/dead fixable stain. Macrophages positive for both CFSE and CellTrace Violet were gated to analyse phagocytosis. **(B)** Phagocytosis of MDCK-HA cells complexed with different pig IgG isotypes, pb27 isotypes (left) and pb39 isotypes (right). Anti-Nipah virus G protein IgG1 mAb was used as a control. Representative data from 3 independent experiments are shown.

We finally evaluated antibody-dependent cellular phagocytosis (ADCP) in which antibody-opsonized target cells activate the FcγRs on the surface of macrophages to induce phagocytosis, resulting in internalization and degradation of the target cell through phagosome acidification. Monocyte-derived macrophages were incubated with each pb27 and pb39 IgG subclasses bound to MDCK-HA cells. Phagocytosis was assessed as the proportion of total viable macrophages that contained material from the labelled target cells (**Figure 7A**). Dose-dependent phagocytosis was detected with all pb27 subclasses except very weak activity by IgG5b and no activity for IgG3. (**Figure 7B**). With the pb39 isotypes, lower ADCP phagocytic activity was induced compared to pb27 with no detectable activity for IgG3, IgG4, IgG5c and IgG5b. This lack of phagocytosis did not correspond with the strong binding of IgG3 to monocyte-derived macrophages (**Figure 4**), suggesting that ADCP might be due to internalisation of immune complexes *via* a different set of receptors.

## 4 Discussion

The *in vivo* mechanism of pathogen neutralization is complex and involves not only the Fab recognition of pathogen epitopes, but also Fc interactions with FcγR bearing cells and other immune components. The balance between activating and inhibitory FcγRs determines the biological effect of circulating immune complexes or antibodies bound to the pathogen or cells. There is no information on porcine Fc function and no anti-porcine IgG subclass specific antibodies are available. For the first time, we have generated eight porcine IgG subclasses of two anti-HA mAbs and defined their Fc-mediated functions. Our results indicate that IgG1, IgG2a, IgG2b, IgG2c and IgG4 all bind well to some cell types and mediate CDCC, ADCC and ADCP. IgG5b and IgG5c exhibited weak binding and variable and poor functional activity. Although immune complexes of porcine IgG3 bound well to monocytes and macrophages and weakly to NK cells, IgG3 did not show any activity in the functional assays (**Table 3**).

**Table 3:** Fc binding to immune cells and Fc effector functions.

Subclass (new)	Subclass (old)	Monocytes	MaPh	NK	CDCC	CD107a Degranulation	ADCP
IgGl	IgGl	++	+++	+++	+++	+++	+++
IgG2a	IgG2a	++	–	++	+++	+++	+++
IgG2b	IgG4a	+++	–	++	+++	+++	+++
IgG2c	IgG6.1	+++	–	++	+++	+++	+++
IgG3	IgG3	+	++	*+I-*	–	–	–
IgG4	IgG6.2	*+I-*	++	++	++	+++	+++
IgG5b	IgG5.1	*+I-*	+	+	–	+	++
IgG5c	IgG5.2	*+I-*	+	+	+	+	++

Among the nine *IGHG* genes, only *IGHG1, IGHG3* and *IGHG4* are believed to be present in all pigs, indicating that these genes are evolutionarily more ancient than the others (10). That these have been fixed across pigs suggests they may have non-redundant functions, whilst the *IGHG2* and *IGHG5* genes may provide some amount of functional variation between haplotypes. *IGHG3* has been suggested to be the ancestral sequence that gave rise to *IGHG1* and *IGHG4* (9, 10, 35). However, the lower hinge regions of IgG3 and IgG1 each share greater sequence similarity to human IgG1 and IgG2, respectively (36). This alternatively suggests that the ancestor of modern pigs (*Sus scrofa domesticus)* had at least two IgG subclasses which expanded and homogenized through gene duplication and conversion. A similar phenomenon has been documented in cattle, sheep, and goats, in which their three IgG subclasses tend to clade by species due to high sequence similarity across the CH domains, but their hinge regions have retained their distinctive sequences and are clearly orthologous between the species (4). Interestingly, the genomic organization and sequence of the pig *IGHG* genes reflects their Fc functional activity: *IGHG3* does not show any functional activity and is localized toward the 5’ end of the constant region, followed by the the weakly functional *IGHG5* genes, and the most broadly and strongly functional *IGHG1*, *IGHG2*, and *IGHG4* genes near the 3’ end.

It is not clear why IgG5b showed a reduced binding and neutralizing activities, more pronounced for the pb27. However, the IgG5b expression vector was constructed using a Lan-1 haplotype sequence which has a deletion leading to a shorter lower hinge region not observed in any of the other subclasses in all haplotypes analysed so far (**Figure 1**) (11). Subclass switching some variable regions to this Fc may conceivably result in altered interaction with the antigen in a V sequence-specific manner. Previous studies implied that the constant region can influence the secondary structure of the antigen-binding site, thus accounting for variation in the magnitude of binding to antigen, fine specificity, and idiotypic recognition observed for subclass switch variants of mAb with identical V domains (37, 38).

Complement activation by pig IgG1 and IgG2a subclasses have been previously reported using guinea pig serum as a source of complement (26). Our results indicate that pig IgG2b, IgG2c, IgG4 and IgG5c, activate complement in addition to IgG1 and IgG2a. IgG3 was predicted to contain motifs most likely to allow complement activation (9). IgG3, unlike other subclasses, is monomorphic and structurally unique; it has an extended hinge, sharing very little sequence homology with other isotypes. IgG3 differs from other isotypes in the CH1, CH2 and CH3 heavy chains domain (9). Furthermore, porcine IgG3 has the potential for three inter-heavy chain disulphide bonds (unlike other subclasses, which have only two), adding more rigidity and space to allow effective complement activation (9). Despite these structural predictions, our results showed that IgG3 did not activate complement. Human IgG1, which is most similar to porcine IgG3 in the lower hinge region, strongly binds human FcγRs, but not porcine FcγRs (36, 39). Conversely, porcine IgG1 is a strong activator of FcγRs, despite sharing the lower hinge sequence motif “VAG-” with human IgG2, which interacts with FcγRs very weakly or not at all (39). Thus, Fc functionality cannot be determined by sequence alone. Further studies are required to investigate why IgG3 fails to activate complement.

The development of a robust NK cell degranulation assay in pigs would be beneficial for characterizing the antibody response to pathogens and to further explore the usefulness of the pig model to test mAb delivery platforms, vaccines, and therapeutics. NK cell degranulation and ADCC in humans are mediated by FcγRIIIa (CD16), which is highly expressed on NK cells and monocytes, and binds IgG1 and IgG3 effectively (2). In pigs, FcγRIIIa is highly expressed on monocytes, NK cells, eosinophils, and neutrophils (12). Porcine IgG1 has strong affinity to FcγRIII, consistent with its high CD107 degranulation and ADCC activity detected in our study (40). The lack of NK degranulation by porcine IgG3 might be due to the lack of interaction with FcγRIII. Because more IgG3 binding was detected on monocytes and macrophages this suggests that it might binds receptors other than FcγRIII. Levels of NK cell degranulation may not correlate with ADCC, which can be mediated by both NK cells with FcγRIII and monocytes with FcγRII (41).

There are several human activating IgG Fc receptors (FcγRI, FcγRIIa, FcγRIIc and FcγRIIIa), an inhibitory receptor (FcγRIIb) and a neonatal Fc receptor (FcRn) (42). There is one high-affinity human Fc receptor, FcγRI (CD64), and two families of low-affinity receptors, FcγRIIa, FcγRIIb and FcγRIIc, and FcγRIIIa and FcγRIIIb (CD16) (2). Both high-affinity and low-affinity FcγRs bind IgG-immune complexes with high avidity, but only the high-affinity FcγRs bind monomeric IgG. Human phagocytic cells such as monocytes, macrophages, neutrophils, eosinophils, and dendritic cells express FcγRI, FcγRII, or FcγRIII and all can mediate immune complex uptake either independently or in conjunction with other FcγRs (43–45). In contrast, the FcγRIIb isoform down regulates FcγRI/IIa mediated phagocytosis and immune complex-induced inflammation (46). In humans, IgG1 and IgG3 have stronger binding affinity to both alleles of FcγRII (R131 and H131) than IgG2 and IgG4 (2). Göttingen minipigs have one high affinity (FcγRIa) and three low affinity (FcγRIIa, FcγRIIb, FcγRIIIa), and a neonatal Fc receptor (12, 47, 48). The two FcγRII isoforms, FcγRIIa and FcγRIIb have shown cross species interaction with human IgG1 and porcine IgG1, binding at similar magnitude, but negligibly to porcine IgG3, which may explain the lack of Fc-mediated phagocytosis by porcine IgG3 (40).

It has been suggested that HA antibody mediated FcγRIII activity is dependent not only on FcγR engagement but also on interaction between HA and sialic acid on effector cells (49, 50). For this reason, antibodies against the RBS or adjacent residues do not induce or only weakly induce Fc-mediated effector function because the second contact point between the RBS and sialic acid is sterically blocked. In contrast broadly neutralising antibodies against the HA stem do not sterically hinder sialic acid binding and trigger ADCC efficiently. Our data is in contrast with these observation as both pb27 and pb39 bind to the receptor binding pocket and their Fc-mediated activity correlates with their affinity and neutralizing activity – the higher the affinity the more efficient the Fc-mediated functions. While we do not yet know if pb27 and pb39 depend on Fc-mediated functions to provide protection *in vivo*, the discrepancy between our results and those of Leon et al, and Cox et al, may at least in part be due to use of very different assays. While we used cytokine activated porcine NK or PBMC and assessed CD107 degranulation or target cell lysis, in studies demonstrating a requirement for HA-sialic acid interaction, Jurkat cells transfected with mouse murine FcγRIV or human FcγRIII were used as effectors and effector activation was measured by bioluminescence (49, 50). Further studies will elucidate whether the location of the epitope on the HA is critical to fully engage the anti-viral activity of innate effector cells *in vivo*.

IgG1 which has the highest functional activity in our studies, has been reported to make up ~40% of IgG in peripheral blood (51). Differential IgG subclass expression and production in the pig are controlled by Type 1 (IFN-γ, IL-12) and Type 2 (IL-4. IL-10) cytokines polarising the response to either cell-mediated or antibody-mediated immunity (52). *In vivo* studies have demonstrated upregulation of, IgG1 by IFN-γ, while IL-10 has been shown to upregulate IgG1 production in porcine B-cells cultured *in vitro* (52). IgG3 comprises >60% of total IgG transcripts in ileal Peyer’s patches (IPP) and mesenteric lymph nodes of late term foetuses and newborn piglets, but expression of IgG3 in the IPP drops in the first 4-6 weeks postpartum. It was speculated that IgG3 may have an important role in the antigen independent-pre-adaptive response in newborn piglets (9). If this is the case, this might explain the lack of activity in our antigen specific assays.

Although this extensive *in vitro* analysis has identified three groups of functionally distinct porcine IgG subclasses, that map to discrete regions within the genomic locus, further *in vivo* assays will be required to confirm the lack of function of IgG3 and the differences between the IgG1, IgG2a, IgG2b, IgG2c, IgG4 and IgG5b and IgG5c. We have already shown that H1N1pdm09 specific pb27 IgG1 and another pb18 IgG1 are protective *in vivo* (23). Altering their Fc regions may potentiate or lessen this effect. However, optimising protective efficacy *in vivo* by Fc-mediated functions might be even more important for broadly neutralizing anti-stem antibodies which appear to require Fc-mediated functions *in vivo* for their efficacy.

## Data Availability Statement

The original contributions presented in the study are included in the article/**Supplementary Material**. Further inquiries can be directed to the corresponding authors.

## Author Contributions

ET, BP, JH conceived, designed and coordinated the study. BP conducted Fc mediated assays and analyzed the data. WM and JCS performed genomic analysis and generated IgG subclasses. AN, AS, JES, MP, SG designed assays, performed experiments and BLI analysis. MP provided project management. AT and PR provided reagents, developed assays for epitope recognition and performed sequence analysis. ET, BP, WM, JCS and JH wrote and revised the manuscript. JH, ET, AT, SG acquired the funding. All authors contributed to the article and approved the submitted version.

## Funding

This work was supported by Bill & Melinda Gates Foundation grant OPP1201470 and OPP1215550 (Pirbright Livestock Antibody Hub); UKRI Biotechnology and Biological Sciences Research Council (BBSRC) grants BBS/E/I/00007031, BBS/E/I/00007038 and BBS/E/I/00007039. AT and PR were funded by the Chinese Academy of Medical Sciences (CAMS) Innovation Fund for Medical Sciences (CIFMS), China Grant 2018-I2M-2-002 and the Townsend-Jeantet Prize Charitable Trust (charity number 1011770).

## Conflict of Interest

The authors declare that the research was conducted in the absence of any commercial or financial relationships that could be construed as a potential conflict of interest.

## Publisher’s Note

All claims expressed in this article are solely those of the authors and do not necessarily represent those of their affiliated organizations, or those of the publisher, the editors and the reviewers. Any product that may be evaluated in this article, or claim that may be made by its manufacturer, is not guaranteed or endorsed by the publisher.
