# Supplementary material for: Fc-Mediated Functions of Porcine IgG Subclasses
Source: Front Immunol. 2022 Jun 9;13:903755. doi: 10.3389/fimmu.2022.903755 (PMC9218351; doi:10.3389/fimmu.2022.903755)
Supplement: Supplementary file 1 [file DataSheet_1.pdf]

Fc mediated functions of porcine IgG subclasses

Supplemental Information

Supplementary Figure 1

A

% Virus neutralization by mAbs at 10 µg/ml									
	X-179A		K163Q (Sa site)		K163E (Sa site)		K130E (Ca site)		Comments
pb39	93	93	100	99	89	103	99	99	Neutralised all variants
2-12C	92	95	100	100	89	100	3	16	Control for K130E
MEDI8852	92	92	100	100	82	102	98	100	Stem mAb control
T26A	92	93	5	15	-3	61	98	99	Control for K163E/Q

B

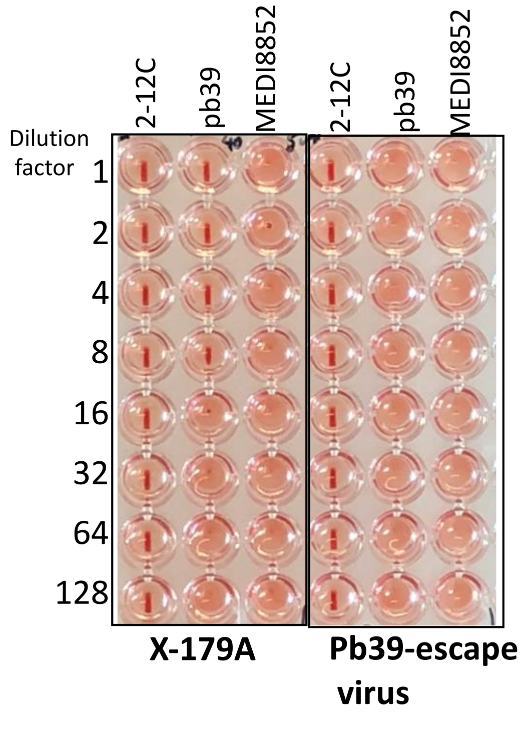

Suppl Figure 1: **A) Virus neutralisation of X-179A and its variants by monoclonal antibodies. B**) Hemagglutination inhibition (HAI) assay. MAbs 2-12C and stem mAb MEDI8852 starting at 5 µg/ml and pb39 starting at 40 µg/ml. Pb39 HAI wildtype virus at 5 µg/ml and do not HAI the escape virus.

Supplementary Figure 2

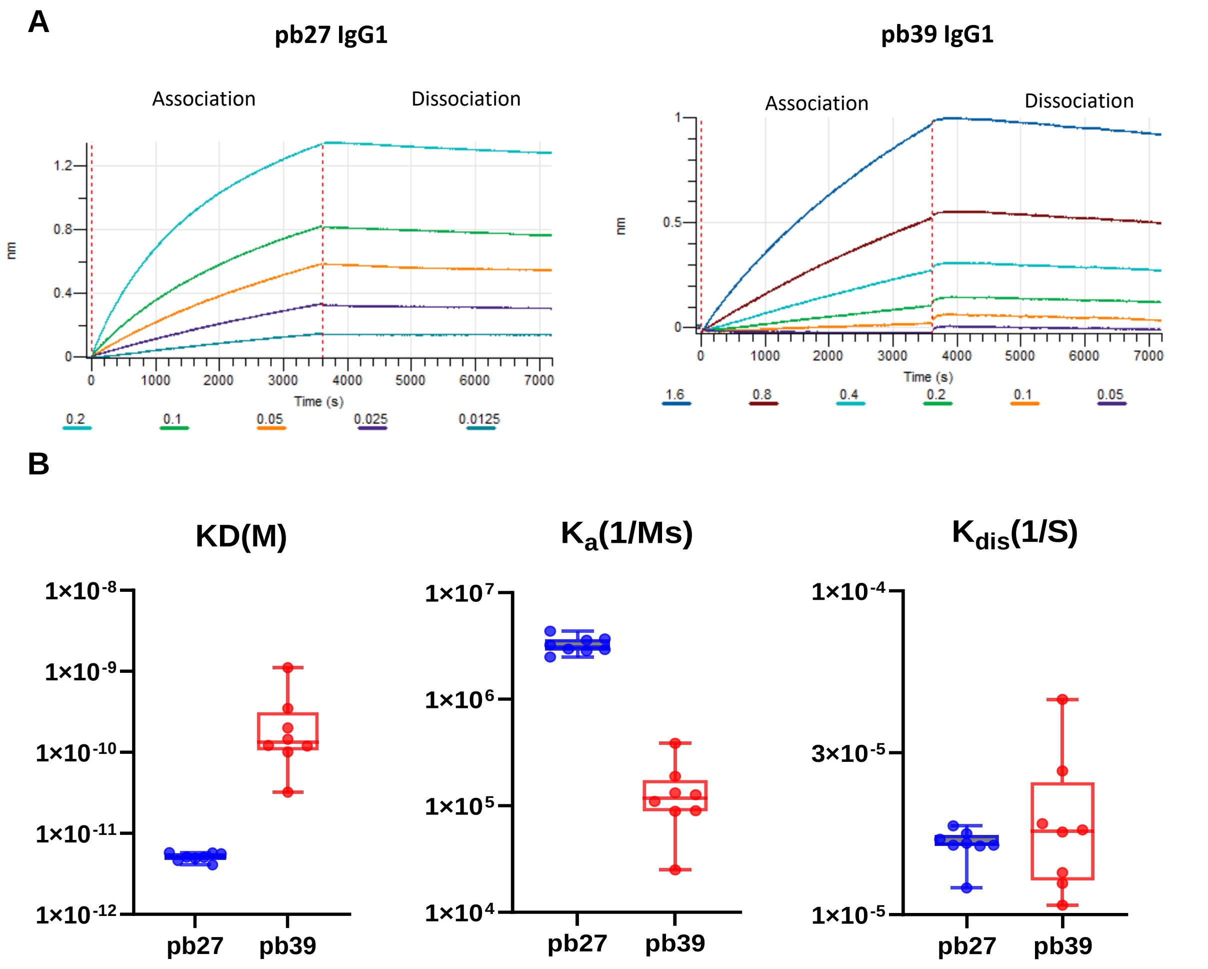

Suppl Figure 2: **Binding characteristics of pb27 and pb39 subclasses as determined by biolayer interferometry**. **A**) Association and dissociation between biosensor-immobilised pb27 IgG1 and pb39 IgG1, and purified virus. Virus concentrations (nM) are shown below each graph. **B**) Binding affinity, association rate, and dissociation rate for all pb27 and pb39 IgG subclasses. Dots show individual subclasses, box the median value and whiskers represent the minimum and maximum value.

Supplementary Figure 3

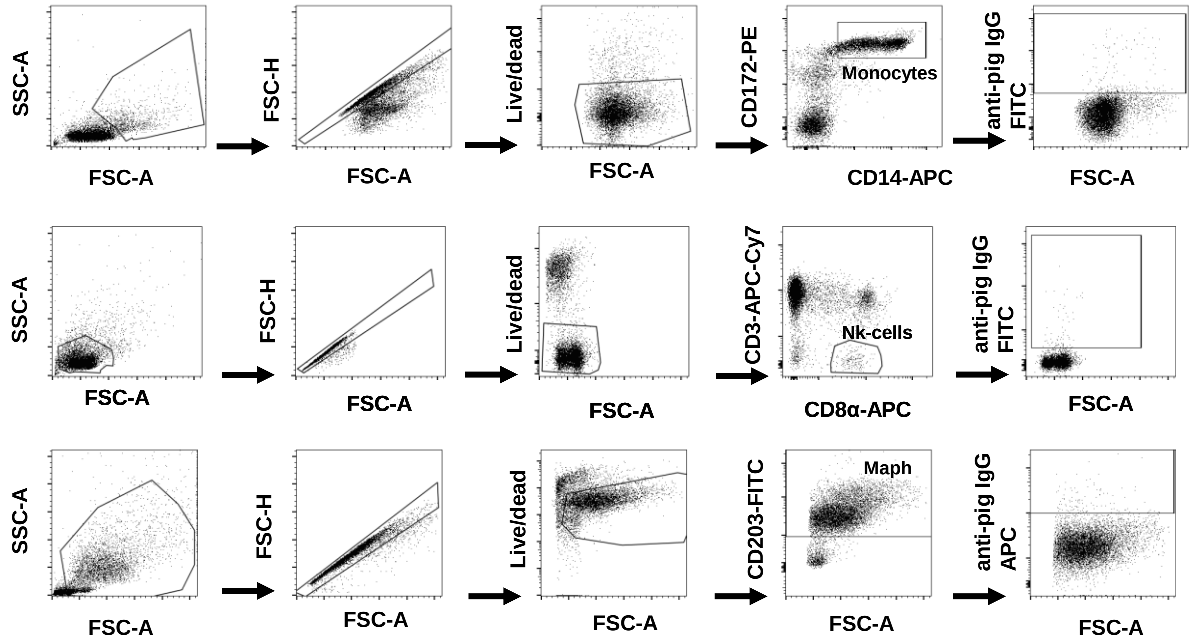

Suppl Figure 3: **Gating strategy for identification of monocytes, macrophages and NK cells**. Successive gates were applied to identify FSC/SSC monocytes, lymphocytes and macrophages, singlet cells, live, CD172a+CD14+ monocytes, CD3-CD8α+ NK cells and CD203+ macrophages.

Supplementary Figure 4

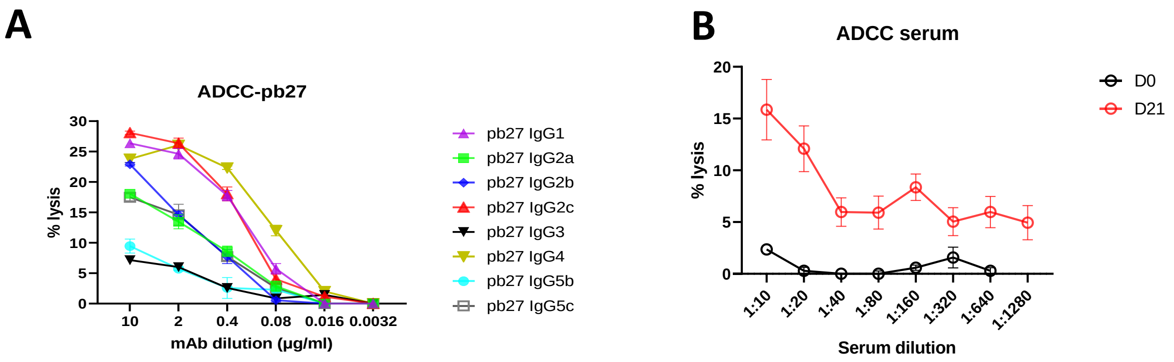

Suppl Figure 4: **Antibody dependent cellular cytotoxicity of IgG subclasses and immune serum**. ADCC activity of pb27 IgG subclasses **(A)** and immune sera **(B)** on MDCK-HA in the presence of overnight stimulated PBMC.
